# Optimizing design of genomics studies for clonal evolution analysis

**DOI:** 10.1093/bioadv/vbae193

**Published:** 2024-12-02

**Authors:** Arjun Srivatsa, Russell Schwartz

**Affiliations:** Ray and Stephanie Lane Computational Biology Department, Carnegie Mellon University, Pittsburgh, PA 15213, United States; Ray and Stephanie Lane Computational Biology Department, Carnegie Mellon University, Pittsburgh, PA 15213, United States; Department of Biological Sciences, Carnegie Mellon University, Pittsburgh, PA 15213, United States

## Abstract

**Motivation:**

Genomic biotechnology has rapidly advanced, allowing for the inference and modification of genetic and epigenetic information at the single-cell level. While these tools hold enormous potential for basic and clinical research, they also raise difficult issues of how to design studies to deploy them most effectively. In designing a genomic study, a modern researcher might combine many sequencing modalities and sampling protocols, each with different utility, costs, and other tradeoffs. This is especially relevant for studies of somatic variation, which may involve highly heterogeneous cell populations whose differences can be probed *via* an extensive set of biotechnological tools. Efficiently deploying genomic technologies in this space will require principled ways to create study designs that recover desired genomic information while minimizing various measures of cost.

**Results:**

The central problem this paper attempts to address is how one might create an optimal study design for a genomic analysis, with particular focus on studies involving somatic variation that occur most often with application to cancer genomics. We pose the study design problem as a stochastic constrained nonlinear optimization problem. We introduce a Bayesian optimization framework that iteratively optimizes for an objective function using surrogate modeling combined with pattern and gradient search. We demonstrate our procedure on several test cases to derive resource and study design allocations optimized for various goals and criteria, demonstrating its ability to optimize study designs efficiently across diverse scenarios.

**Availability and implementation:**

https://github.com/CMUSchwartzLab/StudyDesignOptimization

## 1 Introduction

Recent developments in genomic biotechnology have allowed for increasingly precise characterization and manipulation of genomic, epigenomic, and transcriptomic content at a single-cell level (Li *et al.* 2021). These advances have led to a growing understanding of the importance of somatic variability in normal biological processes—like aging and development—and also disease conditions, like cancer (Olafsson and Anderson 2021). Though genomic medicine is still in its infancy, these technologies offer promise for precise genome monitoring and engineering strategies in the management of these processes (Das *et al.* 2015).

Studies of somatic evolution have been particularly motivated by cancer research, as most cancers are driven by a process of somatic evolution and accumulating variations that gradually give rise to clonal disorder and neoplasias. Cancer cells are typically imbued with one or more of many possible hypermutation phenotypes that produce characteristic patterns of genetic or epigenetic plasticity in their descendants (Loeb 2001). Examples of these processes include chromosome instability (CIN) phenotypes characteristic of TP53 defects (Pino and Chung 2010), point mutation phenotypes of DNA polymerase defects (Barbari and Shcherbakova 2017), or various phenotypes of DNA mismatch repair defects (Peltomäki 2001). Furthermore, neoplasms often contain complex scaffolds of putatively healthy and neoplastic cells, each exhibiting varying genetic and epigenetic changes and spatial distributions within the tumor microenvironment (Martincorena *et al.* 2017). Methods for characterizing processes of somatic evolution in cancers have seen intensive study, but results are highly dependent on the data available to them (Schwartz and Schäffer 2017).

Developing a fuller understanding of somatic evolution and bringing it to translational problems, such as personalized cancer treatment, will hinge in part on our ability to develop rigorous data science frameworks for optimally deploying combinations of the many complementary genomic technologies now available for any given study. In conjunction, numerous statistical and algorithmic methods have been developed to convert various genetic readouts into insight into the population structure and dynamics of cell populations (Ding *et al.* 2014). An ever-expanding set of features we might want to characterize include: cellular phylogenetic trees, mutations (structural, point, and copy number changes) and mutability patterns (mutational signatures), spatial distributions, epigenetic alterations, and transcriptional activity (Grody *et al.* 2023). Researchers seeking to characterize such features have available to them numerous combinations of genomic tools combined with a wide variety of algorithms with which to draw inferences (Pareek *et al.* 2011, Schwartz and Schäffer 2017).

This wealth of options is making ever more complicated the key question of how to design a maximally effective study to gather specific information or test a specific hypothesis about a somatic evolution process within resource constraints. This problem is especially difficult because of the high dimensional study-design optimization space combined with arbitrarily complex cell populations without ground-truth data. Multiomic designs (e.g., combining various sequencing modalities) may be especially powerful for gaining biological insight but are particularly challenging for traditional optimization algorithms (Mangiante *et al.* 2023). Working effectively and efficiently with the complex space of options requires principled alternatives to the current practice of essentially *ad hoc* design. Genomic study design has been relatively unexplored as a target of computational optimization, particularly the challenges of working with ambiguous and heterogeneous biological replicates within the context of large and high dimensional datasets that require extensive computational processing to interpret. One notable exception is work of Tarazona *et al.* (2020), which explored the relationship between multiomic samples and power through the use of Figures of Merit (FoMs) to standardize power calculations across multiomic platforms.

We present a framework to generalize beyond such standardized metrics to arbitrary loss measures and biological instances using iteratively generated real or simulated data. We use a Bayesian active learning approach to balance exploration and exploitation of the sample space to generate study designs that minimize both resource costs (e.g., financial, time, or depletion of biological specimens) and a statistical loss function describing inference quality using a relatively small number of samples. This framework isintended to manage simultaneously a complex design space, even more complex underlying biology, and the inherently stochastic nature of somatic evolution with arbitrary statistical loss measures and datasets. In doing so, we define a generalized study design allocation problem for somatic variation studies under experimental, simulation, and budget constraints. We show that our optimization framework can find effective study designs and guide experimental design strategy for diverse somatic mutability analyses.

## 2 Methods

In order to answer the general study-design problem, we first pose a formal definition of a study design along with a statement of the optimization problem. The following section defines the variables and mathematical framework in detail. The main components of the study design problem are represented by bold text. Somatic evolution instances and their associated genetic study designs are generated by the simulator in Srivatsa *et al.* (2023).

### 2.1 The optimization problem

Our definition requires the following components:

1. In this article, we define a **study design**, **x**, as a vector:
  $$x=\left(\begin{array}{c} rl/fl\\ c\\ 1-e\\ n\\ s\\ \mathrm{Genome}\\ \mathrm{Paired}\\ \mathrm{Informatics} \end{array}\right)$$
  Detailed definitions of each of these variables are found in Table 1; here each of the sequencing variables are condensed into a representative vector for the study design, $x\in R_{0}^{+}{}^{n^{c}}\times Z^{n^{d}}$, where *n^c^* denotes the number of continuous variables and *n^d^* defines the number discrete variables. Many of these variables, like coverage or read length, might be considered discrete, but have a continuous interpretation. Discrete variables may be integer values (e.g., read length) or categorical (e.g., variant caller).
2. We next define a **budget function**$f:R_{0}^{+}{}^{n^{c}}\times Z^{n^{d}}\to R_{0}^{+}$ and a **cost function**$g:R_{0}^{+}{}^{n^{c}}\times Z^{n^{d}}\to R_{0}^{+}$. Informally, we think of the budget function *f* as assigning a cost to a study design (which may be a literal monetary cost of conducting the study or any other measure of resources expended) and the cost function *g* as measuring a fraction of the maximal budget used by the study design. We assume that these two functions are quickly computable for any design **x**. However, the functions may lack a closed form expression and need not be continuous or differentiable.
3. **Constraints** for the design are given by f(x)≤b, where *b* denotes a maximum budget, which in general is a vector of different measures of cost (e.g., money, time, sample usage).
4. A **loss function**$L_{q}(x)$. $L_{q}:R_{0}^{+}{}^{n^{c}}\times Z^{n^{d}}\to R_{0}^{+}$ measures the error with which our study design evaluates properties the somatic system studied. This measure depends on the biology of the somatic cells, described by the *q* subscript in the loss function, where a cell population is generated from a stochastic realization q∼Q(t). The loss function is typically costly to evaluate, requiring computational optimizations applied to potentially many data simulations, and so we seek to minimize the number of real and simulated loss function evaluations needed by our optimization method.
5. A **regularization penalty**$\lambda \in R^{+}$ balances the efficacy of a study design and its resource cost.
6. The **score** of a study design is defined as $\lambda g(x)+L_{q}(x)$, which measures the overall efficacy of a target study design **x**. Note that in our framework, lower scores imply more effective study designs.

**Table 1.** Study design parameters influencing the construction of a research study and associated genetic toolbox.

Parameter	Symbol	Data type	Description
Number of samples	*s*	Integer	Number of distinct tissue biopsies to be drawn from each somatic tissue
Type of sample	*sample*	None	Defines how a sample is taken from the cell population (generally solid or liquid biopsy, though other methods like liquid capture microdissection are less commonly used)
Number of sequencing protocols taken on the *i*th sample	*li*	Integer	Number of individual sequencing protocols taken on the sample *i*
Number of single cells used in a sequencing protocol	*n*	Integer	Number of single cells operated upon under a singular sequencing protocol. If zero, no single cells are generated and only bulk sequencing is performed. Each single cell is sequenced *via* the sequencing protocol defined by the other parameters; e.g., with *n* = 3, we assume that DNA of three single cells are sequenced by a sequencer
Read length	*rl*	Integer—number of bases	Size of reads to be generated from sequencer
Fragment length	*fl*	Integer—number of bases	Defines a superstring from which reads are derived during the sequencing process
Depth/coverage	*c*	Integer—reads per base	Average number of times each nucleotide of the genome is sequenced
Error rate	*e*	Float—fraction of incorrectly sequenced bases	Rate of incorrectly sequenced nucleotides
Paired-end/single-end	*Paired*	Boolean	Binary parameter describing whether the reads are paired end (1) or single end 0. Paired-end reads have two related reads derived from the same fragment
Whole genome/whole exome/targeted sequencing	*Genome*	Boolean	Binary parameter describing whether the sequencer extracts genes from the entire genome (1) or only a subset of the genome 0
Informatics tools	*Informatics*	Categorical/numerical	A parameter describing the type of informatics software used to analyse a sequencing protocol for information. Notably the encoding is flexible, and could be represented many different ways

We are then prepared to define our formal problem statement:

**The Study Design Problem** Given a fixed budget to iteratively generate *M* datasets, ${\left\{X_{q}^{j}\right\}}_{j=1}^{M}$ of the form $X_{q}^{j}={\left\{x_{i},L_{q}(x_{i})\right\}}_{i=1}^{n_{j}}$ corresponding to study design evaluations of a somatic evolution process, we seek to solve the following constrained optimization problem:


(1) $$\underset{x}{\text{argmin}}\;E_{q∼Q}[\lambda g(x)+L_{q}(x)]$$

(2) s.t. f(x)≤b

(3) f(x)≥0

This formulation is realized as a stochastic black-box mixed-integer programming problem. Various approaches have previously been used to solve similar generic optimization problems (Kelley 2002, Liuzzi *et al.* 2012). Some use surrogate functions, either as first-pass solutions or aids in traditional iterative search methods (Gramacy 2020). Others numerically estimate gradients and use these to approach minima iteratively (Spall 2012). Other gradient-free methods instead employ direct search-style algorithms that iteratively explore promising points (Audet and Dennis 2001). Depending on assumptions regarding the function and input domain, algorithms may offer provable convergence guarantees or remain heuristics (Sriver 2004). For reviews of general approaches to related problems see Abramson (2003) and Sriver (2004). The idea of using sequentially simulated instances in combination with diverse biotechnology study design instances is, to our knowledge, new to the somatic evolution literature and there is thus a dearth of methods for the study design problem. Software for more generic optimization is poorly suited to this domain, failing to capture the stochastic and sequential nature of our sampled points, the mixed variable study design vectors, and/or opportunities for parallel computation. The problem is also challenging because typical loss functions are costly to estimate, often discontinuous, and can only be sampled in batches *via* simulation. We thus develop a new approach, borrowing effective ideas from the past literature, such as mesh search for mixed variable programming and surrogate modeling, to create an effective and efficient optimization method.

### 2.2 Optimization strategy

**Figure 1.**
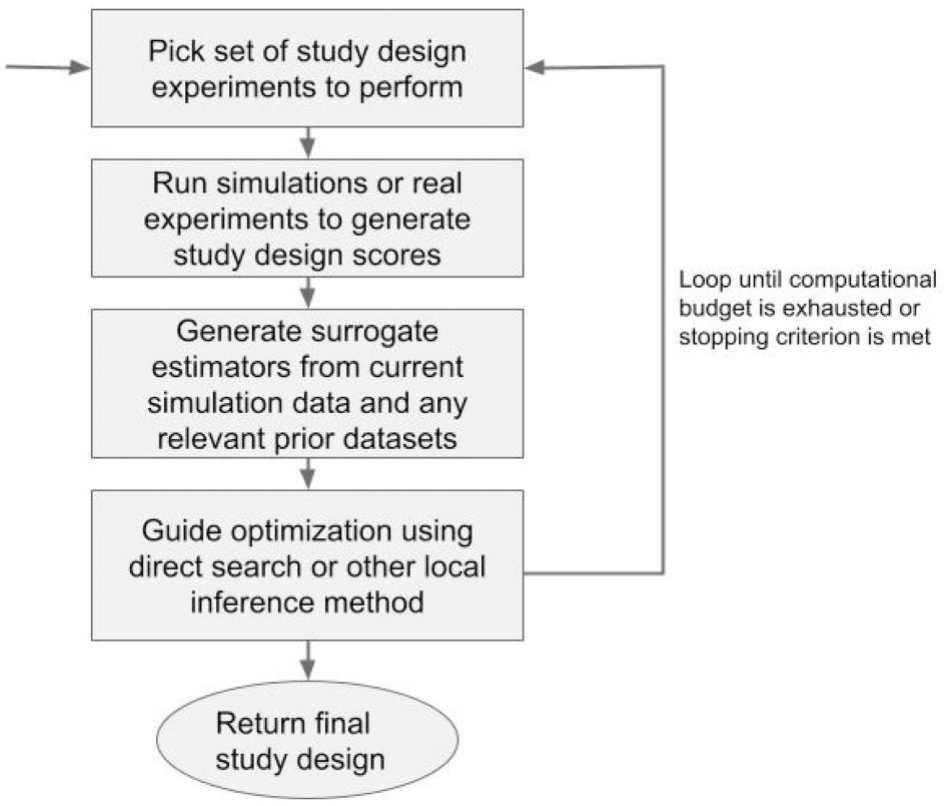
A flowchart of the main algorithm is depicted above. The algorithm repeatedly estimates surrogate functions from simulation calls chosen to explore regions of the search space of uncertain quality while exploiting regions of known high solution quality *via* focused local search.

**Figure 2.**
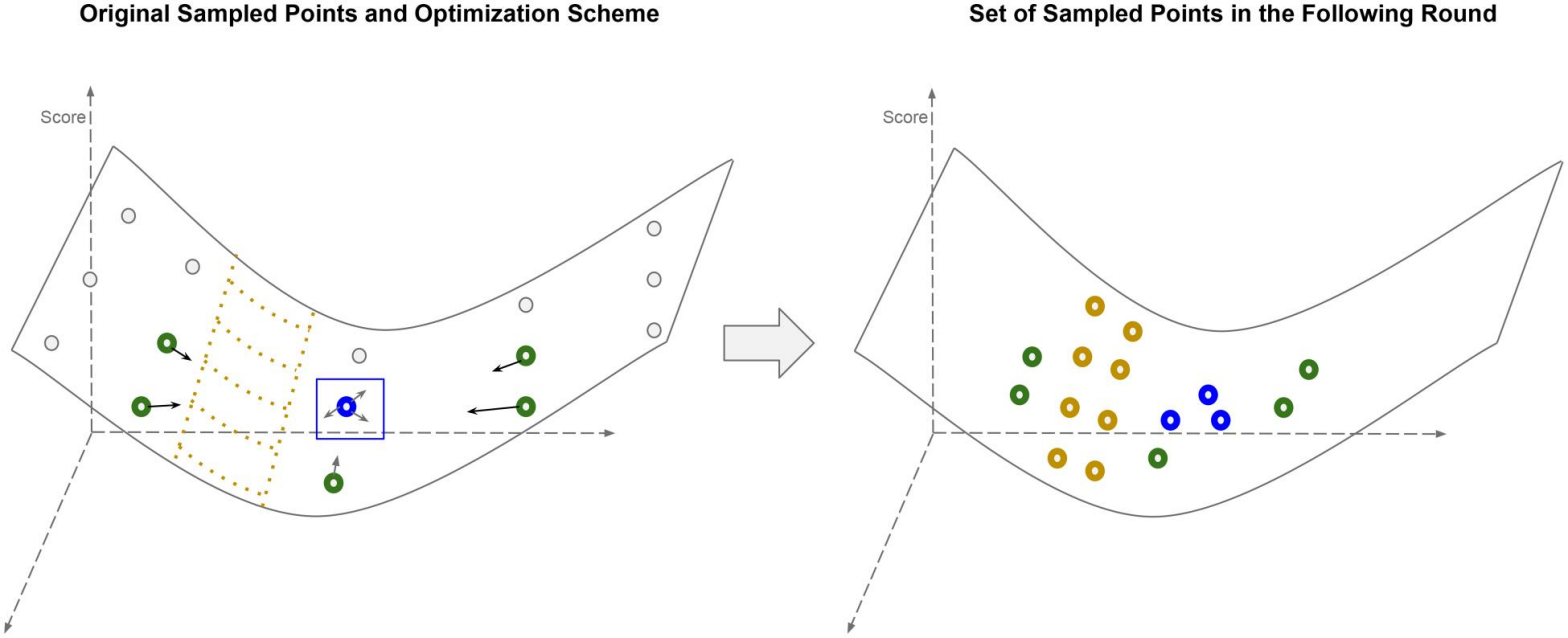
Illustration of the optimization algorithm on a simplified example. The algorithm starts with a Latin hypercube sample of the input space. New points are explored in three main ways to balance exploitation of known high-quality solutions with exploration of poorly characterized regions of the search space. Points depicted in green are generated *via* a gradient descent search step with a numerical estimation of the gradient. Points depicted in gold and by the gold mesh in the left image are drawn from regions of high variance in the Gaussian process estimation and explored in the next round *via* the lower acquisition bound criteria. The minimal point in blue on the left is explored *via* a mesh search of points in the local neighborhood in the next round.

Our overall strategy uses an iterative exploration–exploitation approach to return progressively better solutions to the study design problem despite the high dimension of the search space and computational cost per evaluation. The algorithm first produces a sampling matrix of study designs for both the cost function and loss function, which are then used to build initial surrogate models for the combined cost and loss function. It proceeds by using the surrogate functions to select new points, combining exploration of high uncertainty regions of the sample space with exploitation *via* local neighborhood search around current high performance study designs. After each iteration, the surrogate functions are updated, and a queue of high performance points is iterated based on our latest local and global searches. Cost constraints are maintained by rejecting proposed designs that are over the allowed budget. The algorithm ends when either the simulation budget is exhausted or it fails to find a significantly better point after a fixed number of iterations. Figure 1 provides a flowchart of the algorithm, and Fig. 2 provides a visualization of its basic operation. Pseudocode is provided as Algorithm 1 in Supplementary Section S1.3.

#### 2.2.1 Generating experimental designs

At each iteration, the algorithm must estimate the loss function and potentially the cost function. Here, we assume that we can evaluate each subsequent set of study designs *via* simulation, although one might alternatively integrate the same approach into an automated laboratory (King *et al.* 2009, Holland and Davies 2020). During the initial stage of the algorithm, we lack information about which study designs are most effective. We therefore want a set of study designs that “fills” the domain space and use Latin hypercube sampling (LHS) (McKay *et al.* 2000) for this purpose. Instead of simply sampling from the joint distribution, we partition each study design variable $x^{i}$ (the superscript is used to differentiate between the variable and the data point number) into *n*_0_ equally-sized intervals spanning the range of the variable, where *n*_0_ is the number of samples. We randomly sample from each interval to generate *n*_0_ samples for variable $x^{i}$. This is repeated for each variable $x^{i}$, and samples from each variable are concatenated in a random permutation to generate a full matrix $X_{l}^{0}$ of dimension $n_{0}\times d$. Here, *d* represents the full dimension of a single study design vector **x**, i.e., $d=n^{c}+n^{d}$ the total number of discrete and continuous variables. The set of samples produced by LHS promotes variability in the sample space, but it is still exponentially large. We use the stochastic evolutionary algorithm of Jin *et al.* (2005) to generate a favorable LHS sample *via* a maximin distance. i.e., ${\text{max}\;\text{min}}_{1\leq i,j\leq n_{0}}d(x_{i},x_{j})$ where $d(x_{i},x_{j})$ denotes the (Euclidean) distance between samples. This LHS sample is intended to maximize the minimal distance between any two sample rows in the data matrix, thereby ensuring a more even spread in the data. Since some of these designs may exceed the cost function budget, we reject these samples and generate new ones until the target sample size is exceeded. If the cost function is not obtainable in closed form, we might alternatively approximate this function, similarly drawing samples for a matrix $X_{c}^{0}$. Data matrices $X_{l}^{0},X_{c}^{0}$ are passed to the next stage of our algorithm.

#### 2.2.2 Generating surrogate models

Using our initial data set $X_{q}^{0}$, we then develop a Gaussian process (GP) estimator for our loss function $\hat{L_{q}(x)}\approx E[L_{q}(x)]∼GP(\mu (x),k(x,x\prime ))$. The GP is defined by a mean and covariance function estimating similarity between points in the input space (and encouraging smoothness). We use the Matern 3/2 covariance function:


(4) $$k(x,x\prime )=\sigma^{2}\times (1+\sqrt{3}\theta d(x,x\prime ))\;\text{exp}(-\sqrt{3}\theta d(x,x\prime ))$$

In the above kernel, *d* represents a distance metric and *θ* represents a smoothness hyperparameter. The mean and variance of new sample points can be estimated using a conditional distribution derived from the definition of the Gaussian process (c.f., Rasmussen 2003), i.e.:


(5) $$f(x^{*})|f(X)∼N(k{\left(x^{*},X\right)}^{T}K_{XX}^{-1}f(X),k(x^{*},x^{*})+k{\left(x^{*},X\right)}^{T}K_{XX}^{-1}k(x^{*},X))$$

Here, *X* denotes the set of already observed study design vectors, *K_XX_* the pairwise kernel matrix of our *X* vectors, and $k(x^{*},X)$ a column vector of kernels of $x^{*}$ with each element in *X*. Traditional Gaussian process models can be extended to mixed variables by adapting the kernel distance function (Saves *et al.* 2023), generally factoring it into continuous and discrete kernels with the overall kernel/similarity defined as their product. We use the exponential homoscedastic hypersphere kernel for categorical variables (c.f., Garrido-Merchán and Hernández-Lobato 2020, Saves *et al.* 2023). These functions and inferences are implemented in the Surrogate Modeling Toolbox package (Saves *et al.* 2024).

If we do not have a closed form expression for our cost function g(x), we can also generate an estimator $\hat{g(x)}$ using our dataset $X_{c}^{0}$. We use our Gaussian surrogate function(s) to rapidly compute our overall score function for various study designs: $\hat{T_{q}(x)}=\lambda \hat{g(x)}+\hat{L_{q}(x)}$.

#### 2.2.3 Exploring additional points

A critical step of the overall optimization strategy is generating a new set of study design experiments given previous sets of data ${\left\{X_{q}^{i}\right\}}_{i=0}^{j-1}$. There are three main methods we use to select points: GP acquisition function selection, gradient descent iteration, and mesh search iteration. Given *n_j_* available sampling points for iteration *j*, we define an exploration coefficient, *e_j_*, and generate a large number of points *via* an LHS strategy. The LHS sample is winnowed *via* the lower confidence bound acquisition criteria for GPs. We select the minimum $\left\lfloor e_{j}n_{j}\right\rfloor$ points of the LHS sample to generate for the next iteration *via* the values of the LCB function:


(6) $$LCB_{T}(x)=\mu_{T}(x)-\alpha_{v}\times \sqrt{\sigma_{T}(x)}$$

*α* represents an exploration parameter which emphasizes high variance points. This strategy ensures that we explore “promising” points with low values and/or high variance given our current set of explored points.

The algorithm also explores in the vicinity of the current best points *via* local search in an “exploitation” phase. At the current iteration, the other $n_{j}-\left\lfloor e_{j}n_{j}\right\rfloor$ points are explored *via* local search with *n_m_* dictating the number of points explored *via* mesh search and *n_g_* the number explored *via* gradient descent. The current minimum is designated a “mesh center”, around which a mixed-integer mesh is built. The minimal point can be partitioned into continuous and discrete components, $x=(x^{c},x^{d})$. A mesh is defined by a set of directions, D(x,j), and a “fineness” parameter Δ. D(x,j) is a matrix of dimension $n_{c}\times |D|$, with the columns defining a positive spanning set of the continuous space, $\Theta^{c}$ (informally a set of directions to continue the continuous search). The mesh is defined as:


(7) $$M_{j}(x)=\Theta^{d}\times \{x^{c}+\Delta_{j}d_{j}|d_{j}\in \mathrm{col}(D_{j})\}$$

Informally, we can picture the mesh as a set of lattices surrounding the input point. Each discrete value set, input point, and iteration may have a different lattice. Given we have a fixed simulation budget, we cannot evaluate the entire mesh and instead probabilistically pick from the continuous neighborhood lattice of the current point, the discrete neighbors of the current point, and, potentially, the continuous neighborhood of the discrete neighbors—with each set defined below:


(8) $$C_{j}(x)=\{(x^{c}+\Delta_{j}d_{j},x^{d})|d_{j}\in \mathrm{col}(D_{j})\},$$

(9) $$D_{j}(x)=\{(x^{c},N(x^{d})\},$$

(10) $$E_{j}(x)=\{x^{c}+\Delta_{j}d_{j},N(x^{d})\}$$

We first randomly sample points in the continuous neighborhood lattice ($C_{j}(x)$) of our current mesh center. We then progress to a discrete neighborhood ($D_{j}(x)$) of our current point. For categorical variables one might define such a neighborhood by setting each variable equidistant from one another, while for integer variables (e.g., read length) we can set a fraction of the range as a “discrete ball” surrounding a point. With sufficient budget on the current iteration, we explore the extended continuous neighborhoods of our discrete neighbors $\left(E_{j}(x)\right)$. With a large budget one might exhaust all points in the mesh $M_{j}(x)$.

We perform gradient descent search at the remaining $n_{g}=n_{j}-\left\lfloor e_{j}n_{j}\right\rfloor -n_{m}$ lowest-value points, estimating the gradient numerically *via* the surrogate functions. The next point sampled is:


(11) $$x^{*}=x-\alpha_{g}*\hat{\nabla T(x)}$$

(12) $$\hat{{\left[\nabla T(x)\right]}_{i}}=\frac{\hat{T}(x+\gamma \times e_{i})-\hat{T}(x-\gamma \times e_{i})}{2\gamma }$$

Integer constants *γ* and *α* represent a range of perturbation and movement of the gradient estimator and step (typically both are relatively small). The *e_i_* represents a perturbation vector with a small fraction of the domain range of variable *i* at the *i*th position.

In each case, care must be taken to ensure new points are feasible. For points sampled from our acquisition function, we reject those that violate cost constraints. Within our mesh search, we do not allow for sampling points outside the domain hypercube. If mesh search points violate our budget, we shrink the fineness of the mesh in hopes of finding a smaller local neighborhood where points are feasible. A similar procedure is implemented in each gradient descent step, and we also maintain perturbation parameters such that discrete variables remain integral.

#### 2.2.4 Iteration, stopping criterion, and parallelization

The full algorithm involves iteratively picking new data points to evaluate, evaluating them *via* simulation, and conducting surrogate reestimations of the target function. At any stage after the first iteration, a list of ranked study designs as well as the currently trained surrogate function can be returned to the user. The iteration repeats until the given computational budget is exhausted. At each iteration, the exploration parameter and iterated search parameter can be updated (c.f., Supplementary Table S1). By default, we taper the gradient and mesh search parameters as well as the exploration coefficient with each optimization round; this choice has implications for convergence in theoretical optimization literature (c.f., Audet and Dennis 2001).

### 2.3 Performance considerations

The main factor in run time of the optimization algorithm is the evaluation of the sample design points *via* simulation or experimentation, which typically involves a large fiscal, computational, and/or temporal cost. Denoting *S* as the mean time per simulation batch, our overall run time is therefore proportional to *O*(*MS*), where *M* is our simulation batch budget. The simulation experiments and analysis scripts are readily paralellizable across compute cores and nodes, such that the actual run time is proportional in $O\left(\frac{MS}{YZ}\right)$ where *Y* and *Z* denote the number of cores and nodes respectively. Storage and maximum memory load per machine must also be considered. Due to the large volume of simulated read data generated, our program deletes the genomic data produced after each batch. Thus, the program requires $O(n_{m}G_{m}c_{m}n_{j})$ units of disk space where *n_m_* denotes the maximal number of single cells in all samples, *G_m_* denotes the max genome size, *c_m_* denotes the maximum coverage, and *n_j_* is the number of samples per iteration.

### 2.4 Practical considerations for optimizing an experimental design

In this section, we detail practical considerations an experimentalist might consider when optimizing an experiment using this framework, and also how they might interpret the results from the framework.

The first major consideration is the choice of the cost and loss function. The cost function is intended to model the resource cost of performing a particular study design (where resources might be money, time, samples consumed, etc.). In practice, one might approximate financial costs by fitting a linear regression model to commercial study designs to estimate the cost function at different points in the search space. The loss function is a more nuanced choice for the user that captures the expected value of the results and requires understanding the properties of the somatic system of interest. Researchers might be interested in testing for the presence of a mutation, calling variants, detecting evolutionary structures, and more; the loss function is a way of quantifying how well the study accomplishes the task(s) for which it is designed. Our optimization framework provides flexibility and allows the ability to minimize various machine learning loss metrics like accuracy and recall as well as classical notions of statistical power frequently sought in experimental studies. When evaluating the output scores, notions of a “good” versus “bad” score are dependent on an understanding of both the cost and loss function. If the user chooses to use measures of statistical power, traditional interpretations of *P*-value might be used to evaluate study designs, whereas other loss measures like accuracy or recall for a specific detection task would have their own nuanced interpretations. Comparison of study designs between distinct experiments is generally uninformative, unless similar scoring functions are used across them.

When analyzing the results of the optimization output, the user should observe general trends in the Gaussian surrogate model to observe regions in study design space that appear to perform well by the loss function they have specified. If the computational or experimental budget allows, the user may retest the best-scoring points to ensure their reproducibility or estimate their variance. The user should also consider and analyze the optimization score trends by round to assess whether the algorithm appears to have converged on an optimal solution, or whether there are outlier points that may suggest the design space has not been adequately explored. Finally, in terms of practical experimental selection, the user might choose to map the final lowest cost study designs to the most similar available solutions (e.g., a standard Illumina sequencer setting) or might choose to experimentally construct an *ad hoc* sequencing protocol that the optimization suggests would yield large improvements. Alternatively, some continuous parameters might be converted into discrete parameters, allowing the optimization output to directly map to a set of off-the-shelf sequencing solutions.

Finally, we note that any data-driven approach is highly dependent on the data collected and produced as input for machine learning models. In the ideal case, the user would allow for many rounds of optimization and have the ability to generate scores for a large number of study designs per iteration. Somatic evolution and particularly oncogenic pathways are highly heterogenous. Experimental or computational generation of data should therefore be carefully chosen to mirror the properties (e.g., mutation rates, phylogenetic structure, clonal heterogeneity) of somatic systems of interest.

## 3 Results

**Figure 3.**
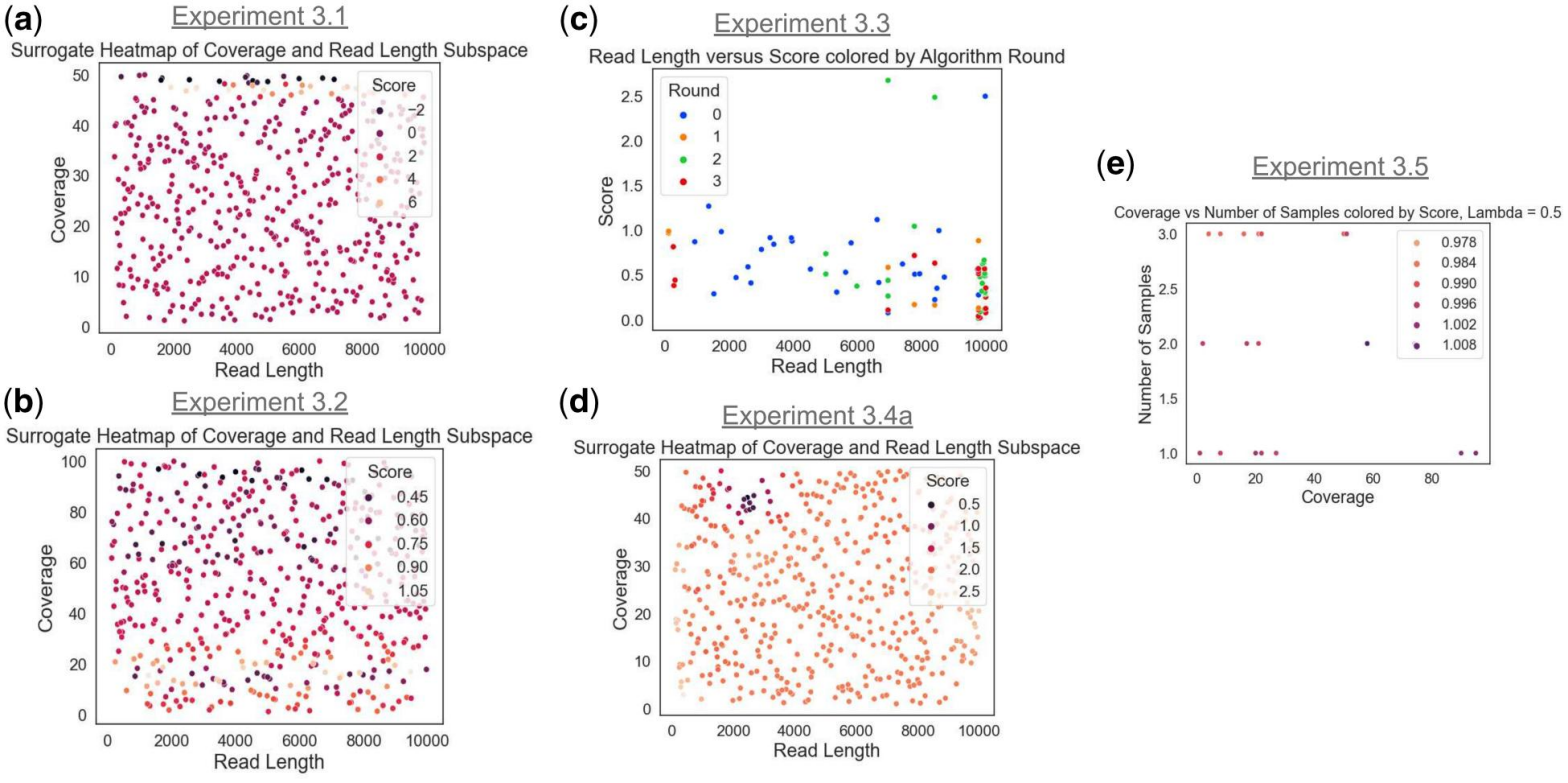
Visualizations from the five experimental queries. (a, b, d) 2D plots of coverage and read length versus score highlighting how different tradeoffs in the study design space by experiment yield different high-scoring regions. (c) Plots read length versus score colored by round of optimization. (e) Plots number of samples and coverage, showing how a higher cost-prioritization favors low coverage high sample points.

The following section demonstrates the proposed method on various hypothetical study design queries. A full study design query is defined by its study design, biology, cost and budget, and loss function parameters. We summarize each use case as it is presented but provide full details in Supplementary Table S3. Similarly, we summarize key results in Table 2 and provide raw best scores in the Supplement as Tables S4 and S5. The main results from each experiment are visualized in Fig. 3, and additional figures for each experiment appear in the Supplementary Materials (Figs S1–S5).

**Table 2.** Summary of experiments and their key findings.

Experiment	Description of experiment	Summary of results
3.1	This was a baseline experiment to determine the best study design to recover accurately SVs and SNVs in somatic tissue	The best study designs tended to occur towards the upper bounds of each parameter searched, which is as expected since these would maximize information content
3.2	This experiment aimed to perform the same query as experiment 3.1—SV and SNV detection accuracy—but with an added cost constraint to each study design to balance a tradeoff between cost and accuracy	The best study designs tended to have read lengths in the middle of the allowed range and were variable in the other parameters, although tending to favor higher coverage and more samples
3.3	This experiment aimed to find a study design best able to quantify a particular somatic evolutionary feature, SV rate	The best designs for this experiment tended to maximize read length and coverage, while other parameters had a more mixed influence on the score
3.4	This experiment aimed to find a minimum fiscal cost study design for measuring SV count to within a target standard deviation	The optimizer seemed to find a “pocket” of search space with very low cost that recovered the variant frequency well, which had read length around 2000, coverage around 25, and two whole genome samples, although the rough search space shows the query is challenging even with a relaxed loss criterion
3.5	This experiment used real liquid biopsy data to optimize a sampling and coverage study design for recovering variants to high accuracy at different cost prioritization	With a higher cost prioritization ( ), the best study designs tended to have lower coverage, whereas with a lower cost prioritization there were more variable study designs, although generally with higher coverage and more samples

### 3.1 Designing a study for maximal mutation discovery

We first assume that we are presented with a tumor and wish to understand its mutational spectrum, i.e., its single nucleotide variations (SNVs) and structural variations (SVs). We assume that we have an effectively infinite experimental budget and that our cost for any study design is thus 0. Resources are encoded here in hard constraints on upper limits, rather than in the objective function. We define the loss function as:


(13) $$L=\frac{SNV_{\mathrm{recall}}+SV_{\mathrm{score}}}{2},\;SNV_{\mathrm{recall}}=\frac{SV_{\mathrm{correct}}}{SV_{\mathrm{actual}}}$$

Calling the SV output locations for chromosome *i*, $C_{i}=\{(a,b)\}$, and calling our ground truth set of SV locations, $D_{i}=\{(c,d)\}$. The SV scoring function is then defined as:


(14) $$SV_{\mathrm{score}}=\sum_{i}\frac{\left|C_{i}+D_{i}\right|}{\sum_{i}|C_{i}+D_{i}|}\sum_{j\in D_{i}}\frac{1(|j\cap C_{i}|>0)}{\left|D_{i}\right|}$$

The SV portion of the loss function measures the fraction of called SVs that overlap with a ground truth variant set. The overall loss function is intended to model the accuracy with which we can correctly detect both structural and point mutations in the genome.

Results for this query appear in Supplementary Table S4 and are visualized in Fig. 3a, with more detailed visualizations in Supplementary Fig. S1. Overall, the best scores tended to be near the upper bound of the coverage parameter and to correspond to the genome sequenced with two samples with additional single-cell draws. Error rate seems to have a negligible effect, which makes sense as we primarily test for recall. Read length seems to negatively affect scores near the lower extremities of the search region. Overall, the results seem mostly to match intuition in this experiment. With an unconstrained and broad-set query, we expect the right tail of most information-containing parameters to be selected, as these represent the sequencing parameters with maximal information, i.e., providing the most sequence data from which to make inferences.

A univariate analysis of the study design space is likely to miss key subspaces that contain high (and low) performing designs. Figure 3a shows trends in certain 2D subspaces of parameter combinations; the coverage and read length combination, on the other hand, shows areas of high and low performance along the coverage axis. A more detailed analysis of further *n*-dimensional subspaces or hypercubes might find subregions of higher performance.

We can also view the action of the optimization algorithm in exploring the design space. A plot of coverage versus score colored by round (Supplementary Fig. S1c) shows that the initial exploration of the parameters is fairly even, but by the final round most samples occur toward the upper end of the permissible range. We also see that in later rounds, some “under-explored” regions of the space are still being searched due to our high exploration parameter. The optimization scores by iteration of the algorithm fluctuate considerably, with a significant number of outlier points. This may be due to noise from the underlying informatics pipeline, biological stochasticity, the high variance sampling strategy, or the relatively low number of points generated per round.

### 3.2 Imposing cost constraints on the design space

We now make the more realistic assumption that a researcher must consider trade offs between study efficacy and resource cost, again assuming that we wish to characterize the mutational spectrum of the somatic cell population. This time, however, we are constrained by a maximum total budget per experiment. We also model our situation by preferring the use of cheaper study designs that recover sufficient signal over designs with higher cost but equivalent efficacy. To model this situation, we create a cost function that linearly and multiplicatively scales with sequencing parameters corresponding to depth and accuracy, intended to theoretically model how costs might scale with different biological parameters. We change the overall score to incorporate a penalty for increased cost. The full scoring function is found in Supplementary Methods Section S1.2. The loss function is identical to that defined in (13). The study design constraints in this experiment were slightly expanded to allow for increased coverage up to 100× depth, and we again replicated independent tumors arising from a stochastic process consisting of independent draws with five subclones and standard mutation rates consistent with tumor literature.

The lowest scoring results are shown in Supplementary Table S4 with results visualized in Fig. 3b and in more detail in Supplementary Fig. S2. As in Query 4.1, the optimal coverage and genome parameters are near their upper bounds (see Supplementary Fig. S2a and b). Unlike the prior query, however, we see that excessive resource usage is penalized. For example, the optimal read length scores tended to be within the middle of the parameter range (see Supplementary Fig. S2c). We might expect multiple-sample study designs to perform best, but we saw that single-sample study designs also performed well. This is possibly because of the multiplicative cost tradeoff associated with additional samples.

We can also see the surrogate function has a different representation with the imposition of the cost function. Figure 3b, a plot of coverage and read length, shows bands of low score interspersed with bands of high score, perhaps signaling dynamic regions balancing cost and loss tradeoff. The exome parameter appears to drive down the score but the paired parameter has a much more negligible effect (Supplementary Fig. S2b). We also can view the action of the optimization algorithm in terms of the points explored. Although there were only two rounds of low-sample optimization, we see that the second round of optimization was concentrated around middle read length values (Supplementary Fig. S2c). It also appears that there is a fair amount of local structure in the points, with nearby points tending to share similar scores suggesting a smoother objective function, as we might expect given that the cost function is deterministic and smooth. Similarly, the optimization scores uniformly decreased in both median and the lower percentiles.

### 3.3 Measuring structural variation rate

A key concept in understanding how somatic mutability influences cancer risk is hypermutability processes. The aging and developing human body is constantly evolving genetically and epigenetically at some basal rates (Li *et al.* 2021, Olafsson and Anderson 2021). In the case of cancers, environmental stresses or damage to key genetic regulators can induce an aberrant somatic evolutionary process that accumulates variations according to characteristic signatures (Alexandrov *et al.* 2020), which may act for many generations following the most recent common ancestor of a tumor population (Körber *et al.* 2023). We model a variant here of the problem of detecting changes in somatic evolutionary processes, seeking a study design that will optimally evaluate the rate of SV accumulation in a tumor. We use a loss function defined by the percentage difference between our called and actual SV counts:


(15) $$L_{q}(x)=\frac{\left|SV_{C}-SV_{A}\right|}{SV_{A}}$$

This loss function is intended to model how accurately we measure the frequency of structural variation without probing the specific SVs. We assume that cost is fixed at 0 and apply similar biological parameters and study design parameter ranges as in the prior experiments.

The best-scoring results are shown in Supplementary Table S4 with visualization in Fig. 3c and in more detail in Supplementary Fig. S3. The highest performing designs for this task generally maximize read length (see Fig. 3c). Coverage also has a positive effect but more weakly effect. The surrogate projections (see Supplementary Fig. S3a and b) demonstrate strong negative correlation with increased read length. These results align with the expectation that high read lengths are critical to SV discovery. Values like error rate did not have a consistent effect on overall score, and the optimizer tended to oversample both the low and high extremes for error rate. A number of outlier points had extremely high mutational count differences, raising the question of whether these points should be considered valid for the optimization or rerun/discounted. Sampling of points from round to round appeared to focus in differing regions of the space (with the exception of the initial Latin hypercube round). Optimization scores trend downwards with each round (see Supplementary Fig. S3c), but are noisy, which might again be due to the small sample size per round.

### 3.4 Generating a minimum viable design for a power hypermutability query

A common question we might ask in the study of cell populations is the presence or absence of a feature characteristic of particular neoplasia. For instance, one might want to know if there is an elevated frequency of a particular class of SV, the amplification of a particular driver gene, or low tumor purity. In each case, a researcher might seek a minimal-cost study design to test the hypothesis that a tissue exhibits a specific property. We model an instance of this problem by assuming that we detected an elevated rate of SVs during initial biopsy and sequencing of a tumor and have now performed surgery and want to check for evidence of a potential relapse by screening new tissue for SV hypermutability. Our query is to find a minimal-cost study design that accurately estimates SV count/rate to within an acceptable range. Here, we design our loss function by assuming that SVs are accumulated by a Poisson process with mean centered around the true count. Then, we check if the cumulative distribution function (cdf) of the observed count lies within a range defined by a percentile difference *threshold* from the median. This choice of loss function is intended to estimate a loss comparing our observed count to the true count based on a notion of statistical power. Assuming here a Poisson distribution of mutation count, we can then assign a notion of likelihood of the observed count under the Poisson distribution. Our primary goal with this loss function is to weight the overall score toward the cost, but only if it fulfills its intended purpose, thus generating a “minimum viable design”. Study design parameter ranges and the biological simulations are kept within standard ranges. The loss and cost functions are defined below as follows, where *SV_C_* and *SV_A_* denote the called and actual number of SVs, and $\Phi^{-1}$ denotes the inverse cdf of a Poisson distribution. We perform two experiments, one with *threshold *=* *0.33 (denoted 3.4a) and *threshold *=* *0.4 (denoted 3.4b) where:


(16) $$L_{q}(x)=\{\begin{array}{cc} 0 & |\Phi_{SV_{A}}^{-1}(SV_{C})-0.5|\leq \text{threshold}\\ 2 & |\Phi_{SV_{A}}^{-1}(SV_{C})-0.5|>\text{threshold}\\ 3 & \text{Caller}\;\text{Error} \end{array}$$

The best-scoring results are shown in Supplementary Table S4 with visualization in Fig. 3d and in more detail in Supplementary Fig. S4. The best-scoring points appeared from highly dissimilar combinations of parameter values. Exploration of our surrogate function (Fig. 3d and Supplementary Fig. S4a–d) showed small and specific regions of the search space as being viable for the query and producing a low score. The vast majority of study designs were not able to pass the “thresholding” criterion created by our loss function. When comparing different threshold values (0.33 versus 0.4), we see a similar pattern of specific local regions of the overall space being effective, with a larger threshold allowing for more regions of the search space to be viable. Overall, the problem of calling structural variant counts was challenging as both thresholds, despite encapsulating a large fraction of the theoretical distribution, saw very few study designs receive a low value. This was to be expected, as calling SVs, especially high frequency layered SVs, is challenging in mixed clonal populations where the frequencies of these variants may not be clonal and are unevenly sampled in the downstream reads. The experiment also shows how a complex query such as determining a minimal-cost study design that fulfills a complicated set of criteria can be posed and solved within our optimization framework.

### 3.5 A variant recall test on a real liquid biopsy dataset with differing cost prioritization

To further validate the efficacy of our study design optimization procedure, we create a test instance on a true cancer genetics dataset—specifically a melanoma liquid biopsy dataset (ENA accession SRP494094, Scaini *et al.* 2024). The spirit behind this optimization experiment is to identify a liquid biopsy schedule to track gene frequency/variation over the course of disease—i.e., in designing a relapse, recurrence, or progression test for a cancer patient—by picking a certain coverage and number of timepoints for blood samples to track frequencies of key driver genes. To make it possible to ask this retrospectively using real data, we frame the problem by considering the entire span of the dataset as hard constraints for the optimization then search for an optimized study design within a subsample of the full data. We define the information content of each full data point as a ground truth. We thus used variant calls from the full set of data as a ground truth and compared those to the variant calls on subsets of the data corresponding to different study designs to define our measure of loss. We mathematically define this in the next paragraph.

We modeled the cost function by increasing financial penalty linearly with coverage and the number of samples, with the maximum of each being 100× coverage and three samples. To bound our cost function between 0 and 1 for a similar range as our loss function, our cost function is thus: $g(x)=\frac{c*s}{300}$. We split the dataset into a training and test set where the training set has 10 patients and the test set seven patients selected randomly. During the optimization procedure, we generate scores on the training portion of the dataset by comparing each subdesign point with the full data associated with that point. For instance, in evaluating a design with coverage 100 and two sample points, we subsample the existing read set for each of the 10 patients in the training set to downsample to coverage 100 and two of the three available blood samples chosen at random. We then generate final scores by running the subsampled data through a variant calling pipeline and comparing the output to the full output generated by the full data. The score is then defined by counting the fraction of the full-data variants recovered by the subsampled data, averaged over all the points in the test set. Denoting $X=\{X_{1},\ldots ,X_{i},\ldots ,X_{D}\}$ to be the full training dataset. Defining *C_i_* to be the set of called variants on the subset data *i*, *Z_i_* the set of variants on the full data point *i*, and D the number of total datapoints used, the loss is then:


(17) $$L(x)=\frac{1}{D}\sum_{i\in D}\frac{1}{\left|Z_{i}\right|}\sum_{k\in Z_{i}}I(k\in C_{i})$$

This loss function is intended to measure what fraction of the total set of variants are recovered by our study design. In the comparison of our optimization results, we generate the scoring function on the test set for the same set of study designs.

Supplementary Table S5 shows a sample of top scores from experiment 4.5. The full optimization is visualized in Supplementary Fig. S5. For a low cost-prioritization parameter lambda, the best scores tended toward the upper end of the number of samples in the training data (see Supplementary Fig. S5a), but were more varied when applied to the validation data. This may be due to the small validation data sample size, but could also suggest that with a small lambda there may be multiple viable designs. With an increased lambda parameter (λ=0.5), the best scores tended to be “low-pass” study designs where low coverage samples could recover most of the variation without incurring additional cost. At this λ=0.5 value, the best scores were often low coverage (see Fig. 3e) but the number of samples was more variable depending on the training or testing set. Finally, this experiment demonstrates a strategy for subsampling to evaluate challenging somatic evolution hypotheses. It also suggests the potential for more sophisticated power tests on study-specific quantities of interest, e.g., if we are interested in minimizing the probability of falsely rejecting a change in blood allele frequency of a target gene set.

## 4 Discussion and conclusion

This article seeks to address a growing problem in genomics: how to make effective use of an ever-expanding repetoire of biotechnological and computational tools so as to balance optimally our ability to discover relevant biology against resource costs so as to deploy finite resources most effectively. We anticipate it becoming a growing concern as well for the practice of genomic medicine, where future cancer treatment will likely involve complex genetic diagnostic and therapeutic strategies, in turn requiring rational design if it is to be used effectively. We develop a general framework for such questions and demonstrate its versatility in a series of simulated case studies, finding effective solutions that align with biological intuition in relatively few rounds of optimization.

The present methods and use cases might be further developed in many ways. We could expand our queries to complex multi-objective loss functions under complex cost constraints. The optimization procedure and solutions might be also tested through ablation studies with modifications of the lambda cost parameter, the exploration parameter, the cost function, the loss function, and budget parameters. We saw considerable noise in some of the study design samples generated, making issues of statistical power also an important area of research. Under certain conditions, provable guarantees or confidence intervals might be shown for the optimal solutions. Further extensions might also include finding conditions for and proofs of efficient convergence given suitable assumptions on the structure and variance of genomic data. Another key finding was that the difficulty of identifying an optimization solution is linked to the complexity of the biological space and of the query itself. Some query functions might be exceptionally smooth, whereas others rough or even discontinuous. The importance of the query to difficulty of the optimization problem also suggests the potential for considering a parallel problem of how to design good queries; this might be an optimization problem in its own right, a matter of education and guidance for users who lack a sophisticated understanding of optimization, or perhaps a human–computer interaction problem of how to design an artificial intelligence (AI) agent to work with a human domain expert to pose their problem most effectively.

The broader use of optimization algorithms in biotechnology study design is relatively unexplored, with numerous opportunities for future work. We anticipate that the same strategy could be adapted for use in conjunction with various other sequencing technologies—including advances in lineage barcoding (Sankaran *et al.* 2022, Weng *et al.* 2024), liquid biopsy (Williams *et al.* 2024), duplex sequencing (Kennedy *et al.* 2014), and high throughput single-cell sequencing platforms (Nawy 2014)—to design highly particular screens in personalized oncology as well as for general somatic evolution studies. Estimation of the loss function and expectation over the biological domain depends on appropriate data collection and sharing strategies. Information sharing—the idea that certain data sets might be used as informative priors in the optimization and selection of new data—could hold promise. The structure of the surrogate function is an important consideration in both the optimization procedure and query analysis. Ideas from semi-supervised or federated learning—where certain sets of data are created through a combination of labeled, un-labeled, and synthetic data from various sources (Wang *et al.* 2013, Zhang *et al.* 2021)—may be of value. Other algorithmic strategies, such as Monte Carlo or variational inference to compute the expectation in (1), may also be of use. The problem also lends itself well to active learning, i.e., adapting a study design optimally as the study proceeds. Prospective experimental and clinical validation studies will be needed to provide further support for this general approach.

## Supplementary Material

vbae193_Supplementary_Data

## Data Availability

The simulated data results for this study are provided with the source code at https://github.com/CMUSchwartzLab/StudyDesignOptimization.

## References

[vbae193-B1] Abramson MA. Pattern Search Algorithms for Mixed Variable General Constrained Optimization Problems. Houston, Texas, United States: Rice University, 2003.

[vbae193-B2] Alexandrov LB , Kim J, Haradhvala NJ et al; PCAWG Consortium. The repertoire of mutational signatures in human cancer. Nature 2020;578:94–101. 32025018 10.1038/s41586-020-1943-3 PMC7054213

[vbae193-B3] Audet C , Dennis JE Jr. Pattern search algorithms for mixed variable programming. SIAM J Optim 2001;11:573–94.

[vbae193-B4] Barbari SR , Shcherbakova PV. Replicative DNA polymerase defects in human cancers: consequences, mechanisms, and implications for therapy. DNA Repair (Amst) 2017;56:16–25. 28687338 10.1016/j.dnarep.2017.06.003 PMC5750057

[vbae193-B5] Das SK , Menezes ME, Bhatia S et al Gene therapies for cancer: strategies, challenges and successes. J Cell Physiol 2015;230:259–71. 25196387 10.1002/jcp.24791 PMC4363073

[vbae193-B6] Ding L , Wendl MC, McMichael JF et al Expanding the computational toolbox for mining cancer genomes. Nat Rev Genet 2014;15:556–70. 25001846 10.1038/nrg3767 PMC4168012

[vbae193-B7] Garrido-Merchán EC , Hernández-Lobato D. Dealing with categorical and integer-valued variables in Bayesian optimization with Gaussian processes. Neurocomputing 2020;380:20–35.

[vbae193-B8] Gramacy RB. Surrogates: Gaussian Process Modeling, Design, and Optimization for the Applied Sciences. Boca Raton, FL, United States: CRC Press, 2020.

[vbae193-B9] Grody EI , Abraham A, Shukla V et al Toward a systems-level probing of tumor clonality. Iscience 2023;26:106574. 37192968 10.1016/j.isci.2023.106574 PMC10182304

[vbae193-B10] Holland I , Davies JA. Automation in the life science research laboratory. Front Bioeng Biotechnol 2020;8:571777. 33282848 10.3389/fbioe.2020.571777 PMC7691657

[vbae193-B11] Jin R , Chen W, Sudjianto A et al An efficient algorithm for constructing optimal design of computer experiments. J Stat Plan Inference 2005;134:268–87.

[vbae193-B12] Kelley CT. A brief introduction to implicit filtering. Technical report. North Carolina State University, Center for Research in Scientific Computation, 2002.

[vbae193-B13] Kennedy SR , Schmitt MW, Fox EJ et al Detecting ultralow-frequency mutations by duplex sequencing. Nat Protoc 2014;9:2586–606. 25299156 10.1038/nprot.2014.170 PMC4271547

[vbae193-B14] King RD , Rowland J, Oliver SG et al The automation of science. Science 2009;324:85–9. 19342587 10.1126/science.1165620

[vbae193-B15] Körber V , Stainczyk SA, Kurilov R et al Neuroblastoma arises in early fetal development and its evolutionary duration predicts outcome. Nat Genet 2023;55:619–30. 36973454 10.1038/s41588-023-01332-y PMC10101850

[vbae193-B16] Li R , Di L, Li J et al A body map of somatic mutagenesis in morphologically normal human tissues. Nature 2021;597:398–403. 34433965 10.1038/s41586-021-03836-1

[vbae193-B17] Liuzzi G , Lucidi S, Rinaldi F et al Derivative-free methods for bound constrained mixed-integer optimization. Comput Optim Appl 2012;53:505–26.

[vbae193-B18] Loeb LA. A mutator phenotype in cancer. Cancer Res 2001;61:3230–9. 11309271

[vbae193-B19] Mangiante L , Alcala N, Sexton-Oates A et al Multiomic analysis of malignant pleural mesothelioma identifies molecular axes and specialized tumor profiles driving intertumor heterogeneity. Nat Genet 2023;55:607–18. 36928603 10.1038/s41588-023-01321-1 PMC10101853

[vbae193-B20] Martincorena I , Raine KM, Gerstung M et al Universal patterns of selection in cancer and somatic tissues. Cell 2017;171:1029–41.e21. 29056346 10.1016/j.cell.2017.09.042 PMC5720395

[vbae193-B21] Mckay MD , Beckman RJ, Conover WJ et al A comparison of three methods for selecting values of input variables in the analysis of output from a computer code. Technometrics 2000;42:55–61.

[vbae193-B22] Nawy T. Single-cell sequencing. Nat Methods 2014;11:18– 24524131 10.1038/nmeth.2771

[vbae193-B23] Olafsson S , Anderson CA. Somatic mutations provide important and unique insights into the biology of complex diseases. Trends Genet 2021;37:872–81. 34226062 10.1016/j.tig.2021.06.012

[vbae193-B24] Pareek CS , Smoczynski R, Tretyn A et al Sequencing technologies and genome sequencing. J Appl Genet 2011;52:413–35. 21698376 10.1007/s13353-011-0057-x PMC3189340

[vbae193-B25] Peltomäki P. Dna mismatch repair and cancer. Mutat Res 2001;488:77–85. 11223406 10.1016/s1383-5742(00)00058-2

[vbae193-B26] Pino MS , Chung DC. The chromosomal instability pathway in colon cancer. Gastroenterology 2010;138:2059–72. 20420946 10.1053/j.gastro.2009.12.065 PMC4243705

[vbae193-B27] Rasmussen CE. Gaussian processes in machine learning. In: Summer School on Machine Learning. Berlin, Heidelberg, Germany: Springer, 2003, 63–71.

[vbae193-B28] Sankaran VG , Weissman JS, Zon LI et al Cellular barcoding to decipher clonal dynamics in disease. Science 2022;378:eabm5874. 36227997 10.1126/science.abm5874 PMC10111813

[vbae193-B29] Saves P , Diouane Y, Bartoli N et al A mixed-categorical correlation kernel for gaussian process. Neurocomputing 2023;550:126472.

[vbae193-B30] Saves P , Lafage R, Bartoli N et al Smt 2.0: a surrogate modeling toolbox with a focus on hierarchical and mixed variables gaussian processes. Adv Eng Softw 2024;188:103571.

[vbae193-B31] Scaini MC , Catoni C, Poggiana C et al A multiparameter liquid biopsy approach allows to track melanoma dynamics and identify early treatment resistance. NPJ Precis Oncol 2024;8:78. 38548846 10.1038/s41698-024-00567-0 PMC10978909

[vbae193-B32] Schwartz R , Schäffer AA. The evolution of tumour phylogenetics: principles and practice. Nat Rev Genet 2017;18:213–29. 28190876 10.1038/nrg.2016.170 PMC5886015

[vbae193-B33] Spall JC. *Stochastic Optimization.* Handbook of Computational Statistics: Concepts and Methods. Berlin, Heidelberg, Germany: Springer-Verlag, 2012, 173–201.

[vbae193-B34] Srivatsa A , Lei H, Schwartz R et al A clonal evolution simulator for planning somatic evolution studies. J Comput Biol 2023;30:831–47. 37184853 10.1089/cmb.2023.0086 PMC10457648

[vbae193-B35] Sriver TA. *Pattern Search Ranking and Selection Algorithms for Mixed-Variable Optimization of Stochastic Systems*. Wright-Patterson Air Force Base, Ohio, United States: Theses and Dissertations, 2004.

[vbae193-B36] Tarazona S , Balzano-Nogueira L, Gómez-Cabrero D et al Harmonization of quality metrics and power calculation in multi-omic studies. Nat Commun 2020;11:3092. 32555183 10.1038/s41467-020-16937-8 PMC7303201

[vbae193-B37] Wang Z , Xu W, San Lucas FA et al Incorporating prior knowledge into gene network study. Bioinformatics 2013;29:2633–40. 23956306 10.1093/bioinformatics/btt443 PMC3789546

[vbae193-B38] Weng C , Yu F, Yang D et al Deciphering cell states and genealogies of human haematopoiesis. Nature 2024;627:389–98. 38253266 10.1038/s41586-024-07066-z PMC10937407

[vbae193-B39] Williams MJ , Vázquez-García I, Tam G et al Tracking clonal evolution of drug resistance in ovarian cancer patients by exploiting structural variants in cfDNA. bioRxiv 2024.

[vbae193-B40] Zhang C , Xie Y, Bai H et al A survey on federated learning. Knowledge-Based Syst 2021;216:106775.

