# Supplementary material for: Optimizing design of genomics studies for clonal evolution analysis
Source: Bioinform Adv. 2024 Dec 2;4(1):vbae193. doi: 10.1093/bioadv/vbae193 (PMC11645549; doi:10.1093/bioadv/vbae193)
Supplement: vbae193_Supplementary_Data [file vbae193_supplementary_data.pdf]

Supplementary Information for “Optimizing Design of Genomics Studies for Clonal Evolution Analysis”

Arjun Srivatsa and Russell Schwartz

S1 Supplementary Methods

S1.1 Programming Implementation and Usage Guide

All code used for the optimization program as well as the raw results can be found at: github.com/CMUSchwartzLab/StudyDesignOptimization. The optimization code is stand-alone in that it can be easily run provided the simulation code is replaced. All required packages can be installed in a conda environment, and the parameters (e.g., budget, cost function, biological parameters, etc.) of the optimization should be changed to best suit the user's query. The full optimization-simulation workflow is tailored to our compute system; as such, efficient replication of the workflow would require changes to the computer code to best suit the user's compute cluster. To fully generate automated results through simulation, a number of genome aligners and variant callers need to be installed as well as modifications to the paralellization scheme and storage containers for reference and output genomes. The simulation of clonal genomes and scientific biotechnology was generated from a slightly modified version of a clonal evolution simulator presented in (Srivatsa *et al.*, 2023). The major aligners used in the experimental section were BOWTIE2 (Langmead *et al.*, 2009), bwa-mem (Li, 2013), and minimap (Li, 2018). The variant callers used were strelka (Saunders *et al.*, 2012) and dysgu (Cleal and Baird, 2022).

S1.2 Cost function for Experiment 3.2 and 3.4

$$g(\mathbf{x}) = (1.3 * \mathbb{1}[Paired = 1] + \mathbb{1}[Paired = 0]) * (\mathbb{1}[Genome = 1] + 0.1 * \mathbb{1}[Genome = 0]) \times (0.03 * s) * (n+1) * [30 * rl + 30 * c + 500 * (1 - \frac{1}{1+\sqrt{\frac{1}{e+10^{-7}} - 1}}) + 2 * c * n + c * rl]$$

S1.3 Algorithm Pseudocode and Informational Parameter Tables

Algorithm 1: Complete Optimization Algorithm Pseudocode

Input: Budget/Cost function; Constraint Bounds and Data Types $\mathbf{l}, \mathbf{u}, \mathbf{c}$; Number of sampling points per iteration n_j; Number of iterations M, exploration coefficient e_j; Maximum cost and regularization parameters c, λ; Mesh, gradient descent, and acquisition function parameters (see Table 1)

Initialize empty data matrix, X, surrogate loss and cost function $\hat{L}, \hat{C}$

Sample initial latin hypercube design of size n_0 while maintaining cost constraints, add to data matrix X

In parallel, simulate points in data matrix X according to the underlying biological problem and generate scores

Train Gaussian process model for loss function (and, if necessary, the cost function) using X

Sort X by score

while $M > 0$ **do**

- Sample Latin hypercube of large size (e.g. $100 * n_j$)
- Get LCB acquisition function values for this sample and pick $n_j * e_j$ new search points based on minimal values and cost constraints
- Generate remaining $n_j - n_j * e_j$ points via mesh search and gradient descent steps using surrogate and current data matrix along with a priori parameter values
- Add new points to X
- In parallel, simulate new points in X according to biological knowledge and generate scores
- Update mesh size and gradient descent parameters
- Sort data matrix X by score
- Update surrogate Gaussian process models using new data point scores
- $M = M - 1$
- Check stopping criterion and **break** if criterion is met

Output: Ranked matrix of study designs, Surrogate model

**Parameters**	**Symbols**	**Description**
Lowerbounds, Upperbounds, Categorical Flags	$\mathbf{l}, \mathbf{u}, \mathbf{c}$	Hard constraints on the range of each parameter and whether they take discrete or continuous values
Experimental Budget, Number of Samples per Iteration	M, n_j	The total experimental budget defines the number of iterations of the algorithm we can perform (i.e., the number of sets of data we can generate) and the number of samples per iteration dictates the total number of individual study designs we can generate per round.
Gradient Descent Step, Perturbation Vector, Scaling Factor, Number of Gradient Descent Sample Points	$\alpha_g^j, \mathbf{e}_j, \gamma, n_g^j$	These parameters define the numerical estimation of the gradient as well as the size of the gradient iteration step. The perturbation vector and scaling factor are used to compute a finite difference equation for the gradient at a certain point, and a new point is generated from the current point and gradient descent step. The number of gradient descent points dictates the number of current minimal points whose gradients are estimated for the next iteration.
Exploration Coefficient, Acquisition Variance Parameter	e_j, α_v^j	The exploration parameter assigns a fraction of points to explore via our acquisition function derived from the Gaussian process as opposed to local search of already promising points. The acquisition variance parameter emphasizes the exploration of high variance points at large values.
Mesh Size, Directional Polling Set, Discrete Neighborhood	$\Delta_j, D_j, \mathcal{N}_D$	The mesh size assigns a range for the lattice search, while the directional polling set and discrete neighborhood definitions assign how we explore local points from the mesh center.
Cost Function, Loss Function	$g(x), L(x)$	The cost function defines the cost for a single study design and can either be given in closed-form to the optimization program or approximated by a surrogate function from data. The loss function describes the efficacy of a study design and must be computed through experiment or simulation.
Maximum Cost, Lambda Regularization Parameter	c, λ	The maximum cost parameter describes the limit in cost of a study design and implicitly assigns a feasible region to our study design space. The lambda regularization parameter balances a trade off between cost and loss in our overall optimization output (i.e., how much emphasis we should place on gains in performance versus increases in cost).

Table S1: Summary of the main algorithm parameters. Parameters like the bounds and cost function indicate user inputs critical to the optimization problem. Technical parameters like the gradient descent step and mesh size impact the action of the algorithm.

**Parameter**	**Symbol**	**Units**	**Description**
Effective Population Size	N_e	Cells	Total cellular population of the region to be sampled. Impacts coalescent times
Number of Clones	k	Clones	Number of distinct genetic somatic cell populations to be sampled
Mutation Rate Lists	M_i	Mutation events per locus per cell division	Lists for each variant class, defining rates per locus per cell division
Mutation Size and Location Distributions	$S, z(x)$	Number of Bases, None	Each mutation type can be tuned over size distributions and single base substitutions can be tuned over signature distributions
Number of Tumors	t	Tumors	Number of distinct sites of somatic evolution to be sampled
Dirichlet Concentration, Clonal Frequency Distribution	α, $g(k)$	None, None	Parameter of a Dirichlet process which is used to derive the concentration of the baseline distribution in a sample. A high value will lead to approximately uniform sampling of clones during sequencing, whereas a low value would favor very uneven clonal frequencies. The clonal frequency distribution is the baseline distribution at high α

Table S2: Biological parameters that influence the simulated somatic evolution process.

S1.4 Experimental Query Settings

Table S3: Experimental query settings - including biological, study design, cost function, and loss function parameters- are described below for each performed experiment.

**Experiment**	**Study Design Parameters**	**Biological Parameters**	**Cost and Budget Parameters**	**Loss Function**
3.1	Study design vectors were generated with parameters from the following ranges:<br>$rl \in [100, 10000]$<br>$c \in [1, 50]$<br>$e \in [0, 0.1]$<br>$n \in [0, 1]$<br>$Paired \in \{0, 1\}$<br>$s \in \{1, 2\}$<br>$Genome \in \{0, 1\}$	Each sample consists of a stochastically generated tumor with 5 clones, with a (relatively) high rate of single nucleotide variants (SNVs); medium for deletions, inversions, and copy number alterations (CNAs); and low for large-scale structural variants (SVs). The genome is a random subset of each human chromosome accounting for 10 percent of a total diploid genome.	All study designs are assigned cost of 0, and lambda weight 0. Our experimental budget is 5 optimization iterations with approximately 20 samples per iteration.	We used a loss function defined by equation 13.
3.2	Study design vectors were generated with parameters from the following ranges:<br>$rl \in [500, 10000]$<br>$c \in [1, 100]$<br>$e \in [0, 0.1]$<br>$n \in [0, 1]$<br>$Paired \in \{0, 1\}$<br>$s \in \{1, 2\}$<br>$Genome \in \{0, 1\}$	Each sample consists of one stochastically generated tumor with 5 clones, with a (relatively) high rate of SNVs; medium for deletions, inversions, and CNAs; and low for large-scale SVs. The genome is a random subset of each human chromosome accounting for 5 percent of the total diploid genome.	All study designs were assigned a cost specified in Supplementary Section S1.2, with lambda weight 1. Our experimental budget was 2 optimization iterations with approximately 20 samples per iteration. The maximal cost of any individual sampled point is set to 100,000.	We used a loss function defined by equation 13.
3.3	Study design vectors were generated with parameters from the following ranges:<br>$rl \in [100, 10000]$<br>$c \in [1, 50]$<br>$e \in [0, 0.1]$<br>$n \in [0, 1]$<br>$Paired \in \{1\}$<br>$s \in \{1, 2\}$<br>$Genome \in \{0, 1\}$	Each sample consists of one stochastically generated tumor with 5 clones, with a (relatively) high rate of SNVs; medium for deletions, inversions, and CNAs; and low for large-scale SVs. The genome is a random subset of each human chromosome accounting for 5 percent of the total diploid genome.	All study designs were assigned the same cost of 0, with a lambda weight of 0. Our experimental budget was 4 optimization iterations with approximately 25 samples per iteration.	We used a loss function defined by equation 15.
3.4	Study design vectors were generated with parameters from the following ranges:<br>$rl \in [100, 10000]$<br>$c \in [1, 50]$<br>$e \in [0, 0.1]$<br>$n \in [0, 1]$<br>$Paired \in \{1\}$<br>$s \in \{1, 2\}$<br>$Genome \in \{0, 1\}$	Each sample consists of one stochastically generated tumor with 5 clones, with a (relatively) high rate of SNVs; medium for deletions, inversions, and CNAs; and low for large scale SVs. The genome is a random subset of each human chromosome accounting for 5 percent of the total diploid genome.	All study designs were assigned a cost specified in Supplementary Section S1.2, with a lambda weight of 1. Our experimental budget was 2 optimization iterations with approximately 30 samples per iteration.	We used a loss function defined by equation 16.
3.5	Study Design Vectors were generated from the following ranges:<br>$c \in [1, 500]$<br>$s \in \{1, 2, 3\}$<br>All other parameters are considered fixed to the real experimental protocol	The biological sample is generated implicitly by the 17 melanoma patients biopsied and sequenced by Scaini *et al.* (2024).	The cost of each study design is defined by $g(\mathbf{x}) = \frac{c*s}{1500}$. We performed 5 rounds of optimization with 30 samples per iteration.	We used a loss function defined by equation 17.

S2 Supplementary Results

This section provides a more detailed visualization of experimental results for each of the five test scenarios, Experiments 3.1-3.5.

S2.1 Experimental Figures

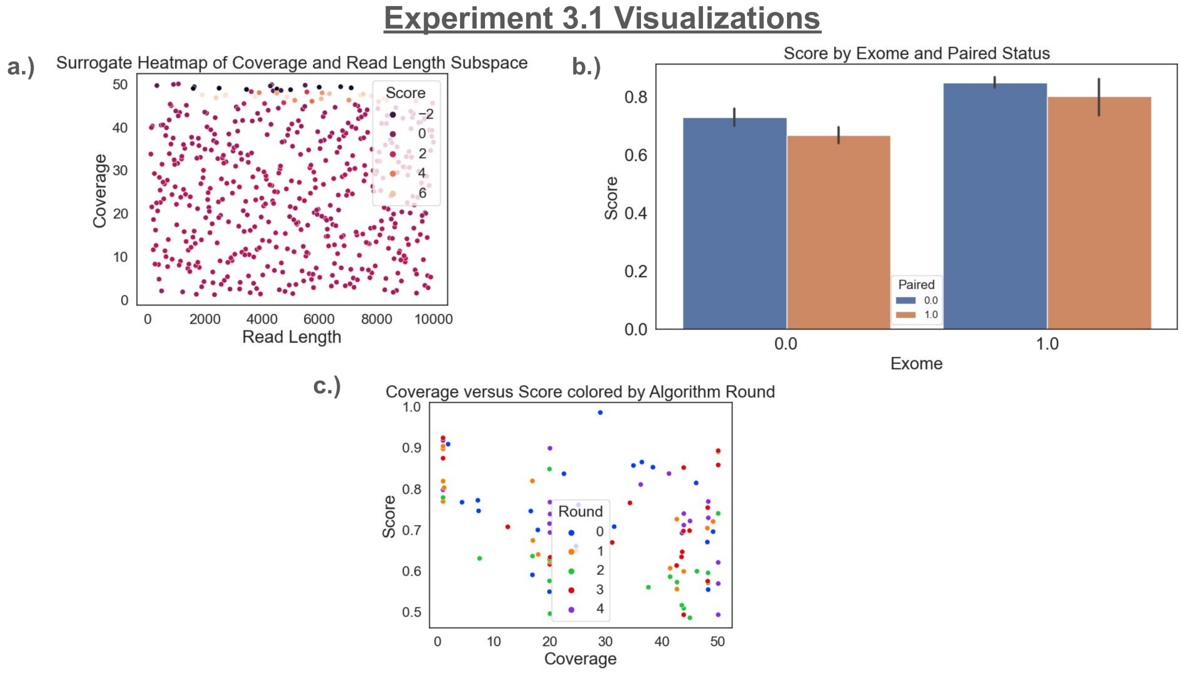

Figure S1: Visualizations for Experiment 3.1, an inquiry into mutation calling accuracy without a cost function. (a) A 2-dimensional surrogate cross section of varying parameters, showing that high coverage values tended to be favored as expected, with read length showing a more ambiguous trend. (b) A parameter bar plot showing how exome and paired sequencing affected scores. As expected, genome sequenced and paired data had the best scores. (c) A single parameter plot depicting how coverage had a negative correlation with score (i.e., higher coverage study designs performed better) and also how the algorithm explored more towards higher coverage values in later rounds of optimization.

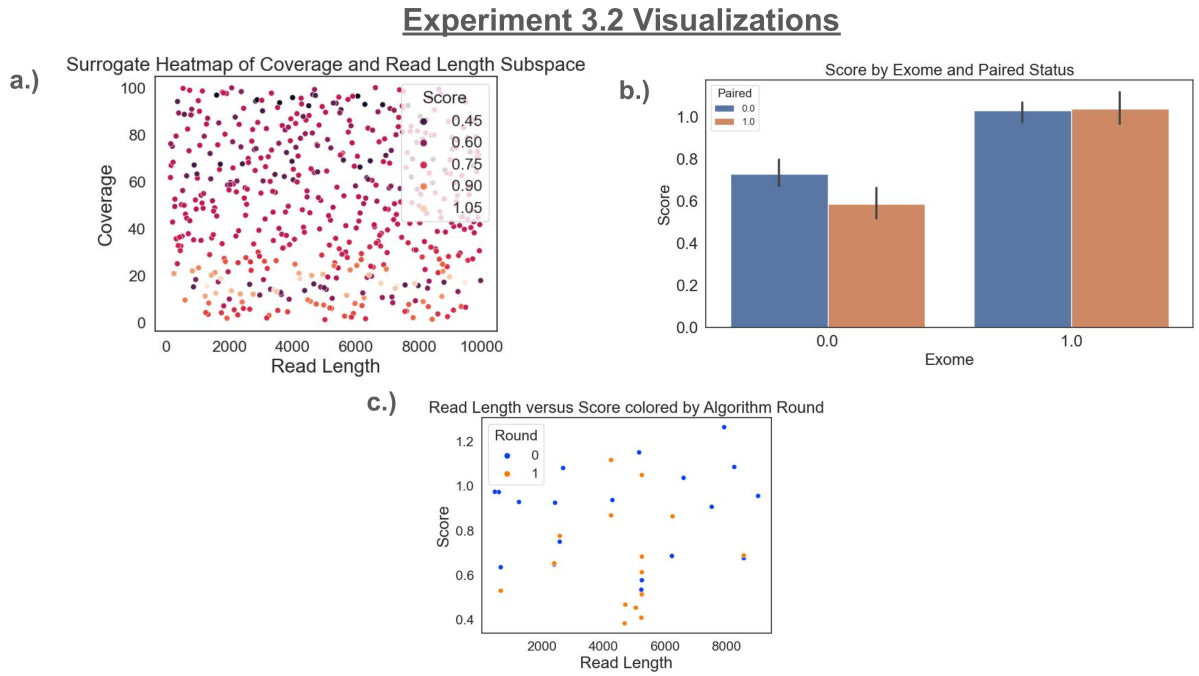

Figure S2: Visualizations for Experiment 3.2, a study design problem combining a cost function with mutational calling accuracy. (a) A 2-dimensional parameter trend plot in coverage and read length showing regions of low score interspersed between regions of high score. (b) A barplot of exome and paired sequencing showing that genome and paired sequencing produced lower scores as expected. (c) Read lengths in the middle of our range are explored more during the second round of optimization and produced lower scores.

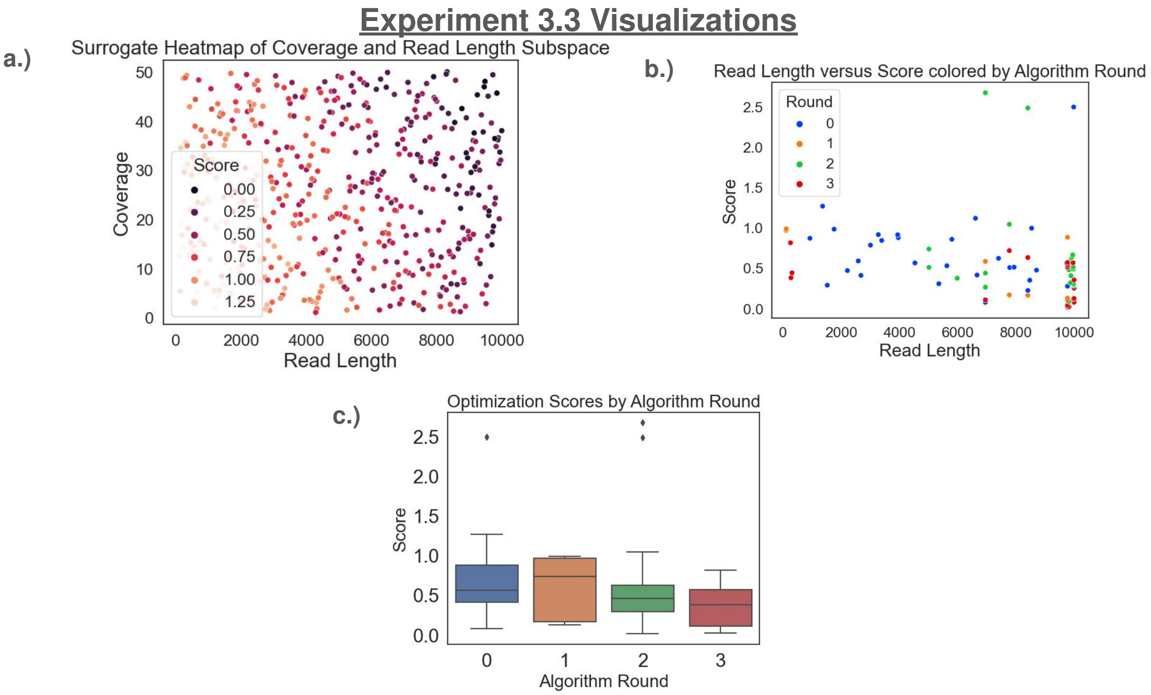

Figure S3: Visualizations for Experiment 3.3, a mutation rate recovery query with no associated cost function. (a) 2-dimensional parameter trends in coverage and read length showing that larger read lengths resulted in better scores. (b) A plot of read length versus score showing negative correlation and exploration by algorithm round. (c) Optimization scores by algorithm round, which shows a decrease in overall score in both mean and minimum by optimization round.

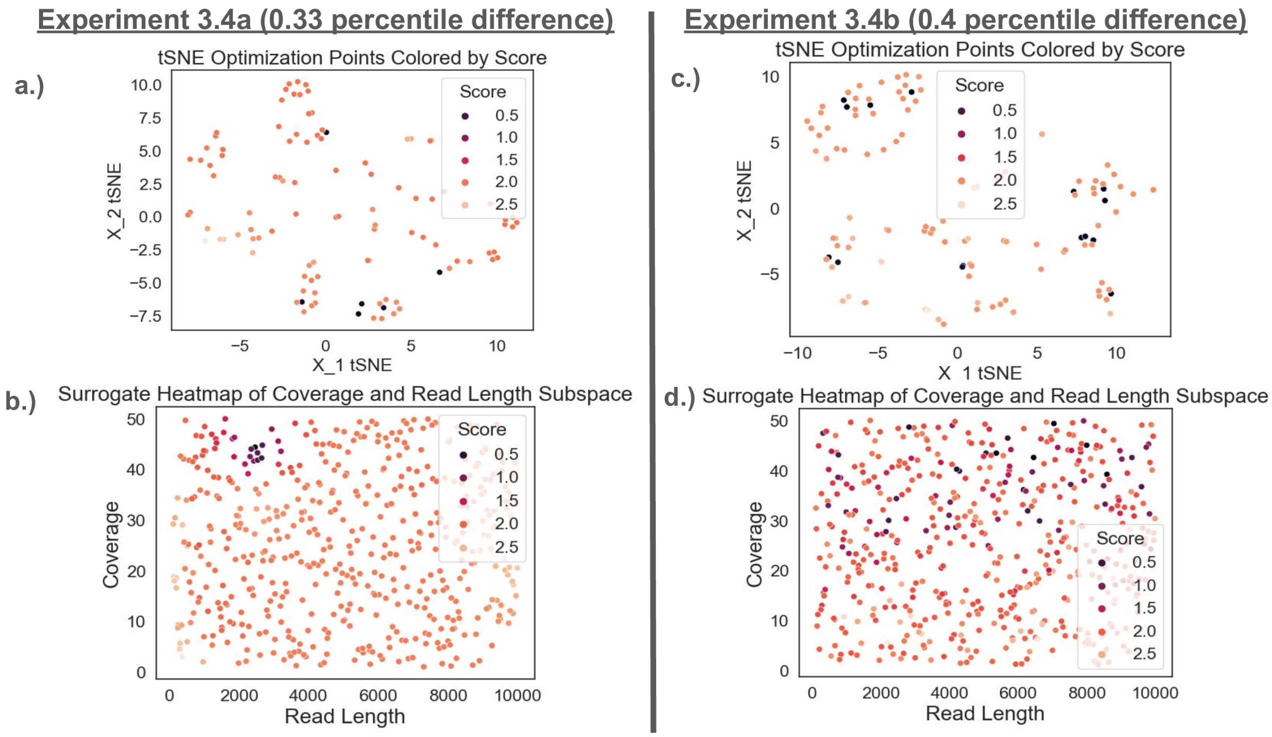

Figure S4: Visualizations for Experiment 3.4, a query designed to detect hypermutable cancer samples at minimal cost. Experiment 3.4a depicts a 0.33 threshold percentile threshold, whereas 3.4b depicts a 0.4 threshold percentile threshold. (a) A tSNE plot of 3.4a depicting how very small regions of the search space yield good scores. (b) A 2-dimensional plot of coverage and read length subspace of 3.4a showing a small region with low score. (c) A t-SNE plot of 3.4b depicting a greater number of subregions with low score compared to 3.4a. (d) A 2-dimensional plot of coverage and read length for 3.4b depicting a larger variety of points with low score.

Experiment 3.5 Visualizations

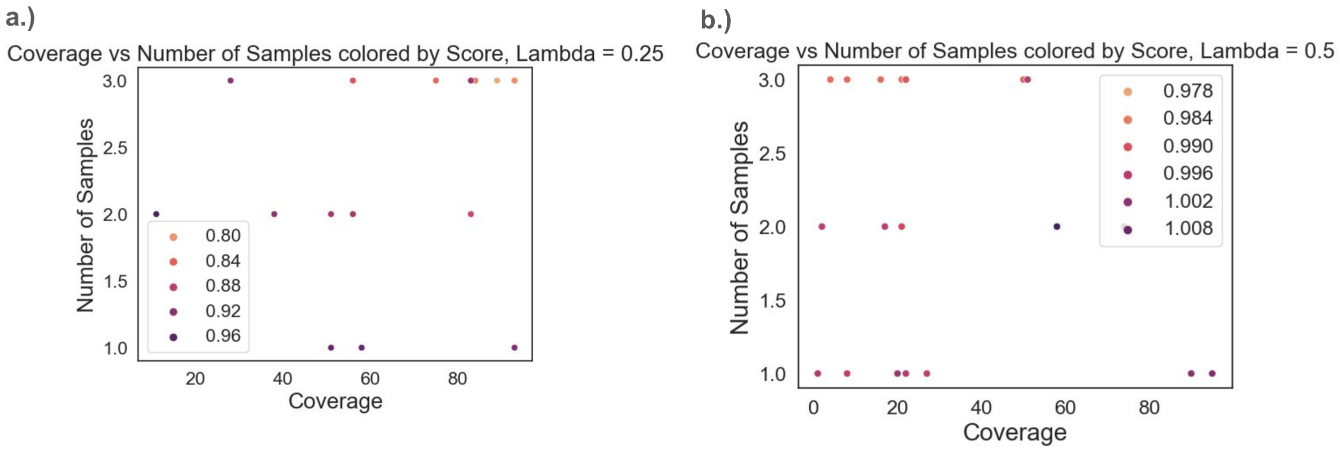

Figure S5: Scores for each study design point on the training data in the coverage and number of sample space for Experiment 3.5. The two plots demonstrate how at lower lambda/cost prioritization the study designs tended to favor high coverage and high number of samples whereas with a higher lambda/cost prioritization, the designs tended to favor low coverage with a larger number of samples.

S3 Results Tables

Best Scores for Experiments 3.1-3.5

Experiment	*rl*	*c*	*e*	*n*	*Paired*	*s*	*Genome*	**Score**
3.1	4904	44.96	0.051	1	1	2	1	0.485
	7471	43.86	0.0	1	1	2	1	0.492
	6023	50.0	0.079	1	1	2	1	0.492
	6022	20.04	0.1	1	1	2	1	0.495
	6306	43.86	0.0	1	1	1	1	0.508
3.2	4690	97.09	0.057	1	1	1	1	0.382
	5232	65.99	0.073	0	1	2	1	0.407
	5055	97.82	0.040	1	1	1	1	0.452
	4713	89.60	0.030	1	1	1	1	0.466
	5250	16.22	0.074	1	1	2	1	0.510
3.3	9775	37.75	0.0	0.0	1	2	1	0.0103
	9826	50.0	0.1	1	1	1	1	0.019
	9775	50.0	0.0	0	1	2	1	0.036
	6951	32.44	0.075	1	1	1	1	0.074
	10000	37.75	0.0	1	1	2	0	0.075
3.4a	581	25.49	0.098	1	1	1	1	0.0103
	7362	36.16	0.013	0	1	1	0	0.020
	2486	22.05	0.026	0	1	2	1	0.021
	2486	23.47	0.028	0	1	2	1	0.102
	2486	45.22	0.1	0	1	2	1	0.105
3.4b	2263	28.57	0.07	1	1	1	0	0.0104
	2263	28.66	0.1	1	1	1	0	0.0105
	2263	37.32	0.050	1	1	1	0	0.012
	2263	49.88	0.080	1	1	1	0	0.014
	792	40.73	0.053	0	1	1	1	0.022

Table S4: The best(lowest) scoring study designs for each of the experimental queries(3.1-3.4) is depicted in the table above.

Best Scores for Experiment 3.5
$\lambda = 0.5$ **(top) and** $\lambda = 0.25$ **(bottom)**

Coverage	Number of Samples	Training Scores	Validation Scores
22.47	3	0.976	0.985
22.48	3	0.984	0.861
26.79	1	0.994	0.836
22.48	1	0.996	0.869

Coverage	Number of Samples	Training Scores	Validation Scores
92.84	3	0.779	0.811
89.36	3	0.784	0.813
27.84	1	0.917	0.646
51.18	1	0.951	0.658

Table S5: The best four study designs with scores on the training and validation set for Experiment 3.5.

References

Cleal, K. and Baird, D. M. (2022). Dysgu: efficient structural variant calling using short or long reads. *Nucleic Acids Research*, **50**(9), e53–e53.

Langmead, B. *et al.* (2009). Ultrafast and memory-efficient alignment of short dna sequences to the human genome. *Genome biology*, **10**(3), 1–10.

Li, H. (2013). Aligning sequence reads, clone sequences and assembly contigs with bwa-mem. *arXiv preprint arXiv:1303.3997*.

Li, H. (2018). Minimap2: pairwise alignment for nucleotide sequences. *Bioinformatics*, **34**(18), 3094–3100.

Saunders, C. T. *et al.* (2012). Strelka: accurate somatic small-variant calling from sequenced tumor–normal sample pairs. *Bioinformatics*, **28**(14), 1811–1817.

Scaini, M. C. *et al.* (2024). A multiparameter liquid biopsy approach allows to track melanoma dynamics and identify early treatment resistance. *NPJ Precision Oncology*, **8**(1), 78.

Srivatsa, A. *et al.* (2023). A clonal evolution simulator for planning somatic evolution studies. *Journal of Computational Biology*.
